# Parasite resistance and the adaptive significance of sleep

**DOI:** 10.1186/1471-2148-9-7

**Published:** 2009-01-09

**Authors:** Brian T Preston, Isabella Capellini, Patrick McNamara, Robert A Barton, Charles L Nunn

**Affiliations:** 1 Max Planck Institute for Evolutionary Anthropology, Deutscher Platz 6, 04103, Leipzig, Germany; 2 Evolutionary Anthropology Research Group, Department of Anthropology, Durham University, DH1 3HN, UK; 3 Department of Neurology, Boston VA Medical Centre and Boston University School of Medicine, Boston, MA 02130, USA; 4 Department of Integrative Biology, University of California, Berkeley, CA 94720, USA

## Abstract

**Background:**

Sleep is a biological enigma. Despite occupying much of an animal's life, and having been scrutinized by numerous experimental studies, there is still no consensus on its function. Similarly, no hypothesis has yet explained why species have evolved such marked variation in their sleep requirements (from 3 to 20 hours a day in mammals). One intriguing but untested idea is that sleep has evolved by playing an important role in protecting animals from parasitic infection. This theory stems, in part, from clinical observations of intimate physiological links between sleep and the immune system. Here, we test this hypothesis by conducting comparative analyses of mammalian sleep, immune system parameters, and parasitism.

**Results:**

We found that evolutionary increases in mammalian sleep durations are strongly associated with an enhancement of immune defences as measured by the number of immune cells circulating in peripheral blood. This appeared to be a generalized relationship that could be independently detected in 4 of the 5 immune cell types and in both of the main sleep phases. Importantly, no comparable relationships occur in related physiological systems that do not serve an immune function. Consistent with an influence of sleep on immune investment, mammalian species that sleep for longer periods also had substantially reduced levels of parasitic infection.

**Conclusion:**

These relationships suggest that parasite resistance has played an important role in the evolution of mammalian sleep. Species that have evolved longer sleep durations appear to be able to increase investment in their immune systems and be better protected from parasites. These results are neither predicted nor explained by conventional theories of sleep evolution, and suggest that sleep has a much wider role in disease resistance than is currently appreciated.

## Background

All mammals studied exhibit some form of sleep, yet the adaptive value of sleeping remains obscure [1]. When viewed from this evolutionary perspective, the time spent in this state of reduced environmental awareness and behavioural quiescence is likely to be costly in terms of predation risk [2-4], competition for resources [2,4,5], and reproductive opportunities [6]. To outweigh these costs, the benefits of sleep must be substantial.

A wide range of hypotheses have been proposed to explain why this seemingly vulnerable and unproductive state has evolved, including suggestions that sleep conserves energy when alternative activities would bring little advantage [7], is required for the consolidation of memories and learning [8], or plays a role in brain development or repair [9,10]. However, phylogenetically controlled analyses investigating the evolution of mammalian sleep durations have produced mixed support for these explanations [3-5], leaving the evolutionary significance of mammalian sleep a mystery.

A further idea that has yet to be tested is that sleep evolved through the need to augment immune defences and protect against disease [11-13]. It is increasingly recognized that the immune system is energetically costly, as evidenced by its impairment under conditions of nutritional stress and when resources are diverted to increased growth or reproductive activity [14]. Since sleep is an enforced period of inactivity and physiological down-regulation, energy that will otherwise be expended during waking activity would be available to meet the demands of the immune system [7,13,15].

This immune theory of sleep is supported by clinical studies that provide evidence of an intimate physiological link between sleep and the immune system. For example, mammals spend more time asleep, and particularly in non-rapid eye movement (NREM) sleep, when infected with a range of parasitic agents (e.g. influenza [16], *Escherichia coli *[13], and *Candida albicans *[13]). Immunomodulatory cytokines – the signalling molecules of the immune system – play a role in both the normal regulation of sleep and its modulation during an immune response [17]. Direct evidence of a role for sleep in immunocompetence is provided by studies showing that rabbits that sleep more following infection have an increased chance of recovery [18], and rats that are totally sleep deprived die with a systemic invasion of bacteria [12]. Finally, the human antibody responses to vaccination can be halved when subjects are deprived of sleep either before or after vaccination [19,20]. While the numerous other physiological changes accompanying sleep deprivation or infection make it impossible to isolate sleep *per se *as a causal factor in experiments [17,21-23], when taken together these studies highlight the potential importance of disease resistance in the evolution of sleep.

**Figure 1:**
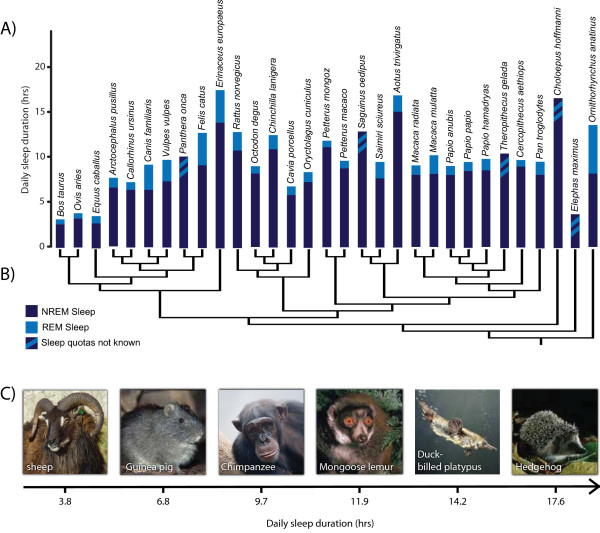
**Variation in mammalian sleep**. (a) Mammals exhibit striking differences in both their daily sleep durations and the amount of time they spend in each of the main sleep phases. Each bar denotes a species specific sleeping time, and the shaded portions show the time devoted to NREM sleep (dark blue) and REM sleep (light blue). (b) The phylogenetic relationship between the species in our dataset. This composite tree was assembled from recently published sources [66-70]. The phylogeny was used to generate independent contrasts [59], and was restricted to the species present in immune investment and infection status analyses. The available data on sleep durations could not always be matched to both haematological and parasite data, thus, some species were only represented in one of the analyses. (c) A selection of species within the dataset, showing the wide-range of sleep durations that have been recorded. Photo credits: B. T. Preston, The Max Planck Institute of Evolutionary Anthropology & Arco Images.

Here, we assess the evolutionary relationship between sleep and immunocompetence across a wide range of mammalian species by examining the extensive variation in sleep times (between 3 and 20 hours a day [1]; Figure 1), investment in immune defences, and parasitic infections.

## Results and Discussion

We first assessed the influence of sleep on the immune system. To do so we extracted data on sleeping times for different mammalian species from the published literature and matched these data where possible with white blood cell counts reported by the International Species Information System (ISIS [24]; [see Additional File 1]). We use white blood cells as a proxy for immune system investment as they are central to all immune responses and are a measure of immunocompetence [25,26]. White blood cells originate in bone marrow and are derived from the same hematopoietic stem cells that produce red blood cells and platelets [25]. As these latter cells have no direct immunological function, we use them as natural controls to test the specificity of any relationship between sleep and the immune system. If a key selective advantage of sleep is that it allows greater investment in the immune system, then species that sleep for longer should have increased numbers of immune cells in circulation, but there should be no similar relationship with control cells. After matching species values from each database we were able to analyze data for 26 mammalian species while controlling for confounding factors (body size and activity period; see the Methods for details).

**Figure 2:**
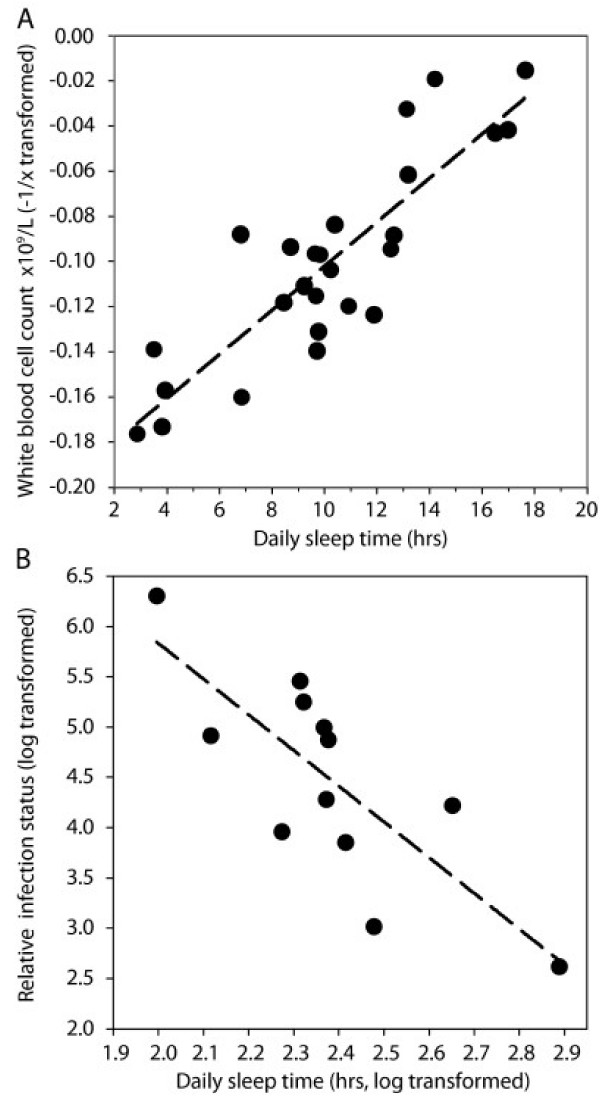
**Sleep, immune defences and parasitism**. Interspecific evidence that sleep protects against parasitic infection. (a) The number of white blood cells in peripheral blood increases among species with longer sleep durations. The fitted line is derived from a multiple regression and controls for a significant influence of body mass and activity period. (b) Species that sleep for longer are infected by fewer parasites. Relative infection status takes account of both the number and prevalence of different parasites infecting host species and corrects for differences in sampling effort [29-31].

As expected if sleep enhances immune defences, species that engaged in more sleep had higher numbers of white blood cells circulating in peripheral blood (coefficient = 0.00976, s.e. = 0.00171, t_22 _= 5.71, *P *< 0.001; Figure 2a). Across our dataset, a 14 hour increase in sleeping times corresponded to an additional 30 million white blood cells in each millilitre of blood (a 615% increase). Crucially, no similar patterns were evident with either red blood cells or platelets (red blood cells: coefficient = -0.028, s.e. = 0.113, t_24 _= -0.24, *P *> 0.8, platelets: coefficient = -0.00602, s.e. = 0.00576, t_21 _= -1.04, *P *> 0.3).

We also tested predictions using phylogenetically independent contrasts to account for the lack of statistical independence in species data [27]. These analyses examine evolutionary change and showed that when lineages evolved longer sleep durations, they also increased their white blood cell counts (coefficient = 0.0153, s.e. = 0.00250, t_23 _= 6.11, *P *< 0.001). Again, this relationship was specific to immune cells (for red blood cells: coefficient = -0.200, s.e. = 0.0123, t_23 _= -1.63, *P *> 0.11, for platelets: coefficient = -0.00463, s.e. = 0.00628, t_21 _= -0.74, *P *> 0.45), leading to an increase in the ratio of immune cells to other blood cell types when species evolved longer sleeping durations (coefficient = 0.0639, s.e. = 0.0108, t_20 _= 5.93, *P *< 0.001).

Total white blood cell counts are a compound measure of the abundance of different cell types, each of which fulfils specialized immunological roles [25]. We therefore also investigated whether the correlated evolution of sleep and white blood cell counts was limited to specific cell types. Neutrophils constitute the largest component of the innate immune system, representing 47% of white blood cells in our sample, and are seen as a first line of defence that responds rapidly upon detection of invading pathogens [25]. Analysis of independent contrasts indicated that higher numbers of neutrophils in the bloodstream have evolved in association with elevated sleep durations (bootstrapped coefficient = 0.0948, s.e. = 0.0223, n = 25, P < 0.001). Lymphocytes, which account for 44% of the white cell count in our sample and are mediators of the acquired immune response [25], were also present in greater numbers when evolutionary increases in sleeping durations occurred (bootstrapped coefficient = 0.0103, s.e. = 0.00385, n = 25, P = 0.004). Finally, eosinophils and basophils, which are relatively minor components of the white blood cell count (5% and 1%, respectively) and act primarily against macroparasites [25], showed the predicted association with sleep (eosinophils: bootstrapped coefficient = 0.0246, s.e. = 0.0125, n = 25, P < 0.035; basophils: coefficient = 0.0676, s.e. = 0.0198, t24 = 3.42, P = 0.002). Only monocytes, accounting for 5% of the total white cell count [25], failed to show a significant association with sleep durations (bootstrapped coefficient = 0.0478, s.e. = 0.0323, n = 25, P > 0.14). Thus, evolutionary increases in the abundance of four of the five immune cell types have occurred in association with longer sleep times, suggesting that increased sleep may allow a generalized heightening of immune defences.

Next, we addressed hypotheses regarding the role of the two main sleep stages (NREM and REM) in immune system investment. In clinical studies, increased duration and intensity of NREM sleep during illness are associated with an improved prognosis, but occur at the expense of REM sleep [11,13,18]. From this, it is argued that immunological benefits of sleep occur while brain function is down-regulated during the NREM phase [11,22]. Our results did not support this suggestion, however, as we found that evolutionary increases in both NREM and REM sleep occur in parallel with elevated white blood cell counts (NREM: coefficient = 0.0853, s.e. = 0.0249, t_17 _= 3.42; *P *= 0.003; REM: bootstrapped coefficient = 0.0633, s.e. = 0.0112, n = 19, *P *< 0.001). Thus, evolutionary increases in sleep are associated with increased investment in the immune system regardless of its specific form.

Finally, we assessed whether a role for sleep in enhancing immune defences could translate into improved resistance against parasitic infections. We were able to match sleep times with parasitism for 12 mammalian species from the *Global Mammal Parasite Database *[28], which details the diversity and prevalence of microparasites (viruses, bacteria and fungi) and macroparasites (helminths, protozoa and arthropods) that infect wild populations of mammals [see Additional File 1]. If sleep is effective in protecting against infection, then species that engage in more sleep should have fewer parasites (measured as a combination of species richness and prevalence, see Methods). After correcting for differences arising from sampling effort [29-31], we found this predicted relationship (coefficient = -3.554, s.e. = 0.888, t_10 _= -4.00, *P *= 0.003; Figure 2b). This analysis suggests that across the 10 hour range of sleep durations present in the dataset there is a 24-fold decline in levels of parasitism. A significant negative relationship was also evident in analyses of independent contrasts (bootstrapped coefficient = -3.48, s.e. = 1.51, n = 11, *P *= 0.006). Thus, as species evolved longer sleep durations and enhanced their immune systems, they become less parasitized.

Our results are consistent with parasite resistance having played an important role in the evolution of sleep, and suggest therefore that sleep is of greater immunological significance than is currently recognized. It had been noted that the physiological links between sleep and immunity that have been highlighted by experimental studies (e.g. in rats [12]) could arise from a negative impact of sleep deprivation on the brain, leading to an impaired coordination of immune system activity [11,17]. However, our findings reveal strong relationships between sleep and immune defences in the absence of sleep deprivation, and thus point to a more direct constitutive role for sleep in promoting immunocompetence.

We suggest that sleep fuels the immune system. While awake, animals must be ready to meet multiple demands on a limited energy supply, including the need to search for food, acquire mates, and provide parental care. When asleep, animals largely avoid these energetic costs, and can thus allocate resources to the immune system (*sensu *[13,15]). Unlike the energy conservation hypothesis of sleep [7], this reallocation hypothesis predicts little or no overall energy savings during sleep. This appears to be true: in humans, for example, energy savings from eight hours of sleep would be expended within one hour of waking (63 kilocalories [32]). Direct estimates of the energetic cost of maintaining immunity are not currently available. However, numerous studies have suggested that these costs are large enough to generate trade-offs with key life history traits, such as growth and reproduction (e.g. [33,34]). Increased energy requirements when the immune system is upregulated also point toward a substantial metabolic cost associated with immune defence. Even during mild antigenic challenge, basal metabolic rate can be increased by as much as 15 to 30% [14,35-37]. Thus, a generalised elevation of immune defences may come at considerable energetic cost.

The energetic costs of immune system maintenance and routine functioning take multiple forms (see [14]). These include the relatively short lifespan and so high turnover rate of granulocytes (every 2 to 3 days [25]), the cost of sustaining the hypermetabolic rate of immune cells [38], and repairing the immunopathological damage that results when cytotoxic compounds are released by immune cells responding to antigens [39]. Thus, an influence of sleep need not be confined to investing in greater numbers of immune cells. Indeed, both antibody responses and natural killer cell activity are reduced following sleep deprivation [19,40], showing that sleep could have a far broader influence on immunocompetence.

Our results and interpretation are consistent with experimental studies showing that animals sleep for extended periods when mounting an immune response [13,16]. If evolved increases in sleep allow animals to channel more energy into their immune defences and so protect against the development of acute infections, then short term increases in sleep may help provide the additional energy required for an acute phase response to an already established infection [13-15]. The possibility also exists that evolutionary and facultative changes in sleep share a common underlying mechanism. Short term increases in sleep appear to be triggered by immunomodulatory cytokines that are released by white blood cells during immune reactions [17]. If larger numbers of white blood cells produce a greater immune response to antigenic challenge, and hence a greater release of sleep promoting cytokines, this could potentially drive evolutionary increases in sleep durations.

Our finding that both sleep phases are associated with immune investment appears to be in conflict with observations that REM sleep is reduced during acute infection (e.g. [13]). It should be noted, however, that the advantage gained through evolutionary increases in normal sleep can also differ from the selective advantage of modulating sleep phases during the course of an infection. For example, REM sleep is partly characterized by a loss of thermoregulation [41], and thus it is argued that REM sleep is inhibited during an acute phases response to infection as it would prevent animals from maintaining an elevated body temperature that impedes further microbial proliferation [42]. While this explanation is plausible, it cannot be applied to evolutionary changes in normal REM sleep durations and immune investment, which occur in the absence of an acute phase response. As data become available on sleep architecture during the acute phase response of different species, comparative studies may be able to assess the benefits of REM sleep suppression directly. Similarly, analysis of the increased intensity of sleep that occurs during infection, as identified through an elevation in slow wave activity [22], could reveal an additional role of the 'quality' of sleep.

It is commonly suggested that sleep may serve multiple functions (e.g. [1]). While our analyses yielded no evidence that sleep influenced cell production in the other physiological systems we assessed, they do not eliminate the possibility that sleep could have an additional function(s) elsewhere in the body. In particular, it has been argued that sleep is 'primarily for the brain' [43], which is a view that has both intuitive merit and considerable experimental support (see review [44]). However, from an evolutionary perspective, phylogenetically controlled analyses have yet to produce support for a key expectation of this hypothesis, namely that species with greater cognitive abilities should require more sleep. At best, a recent study has suggested that REM sleep durations may increase with the brain size of mammals ([3] but see [4]). Since REM sleep usually accounts for less than 20% of total sleep durations [our dataset; see Additional File 1], these comparative findings cannot explain why different mammalian species sleep for as long as they do. Instead, it may be that sleep quality is of greater importance to brain function than the overall duration of sleep *per se*, which is a possibility that could be assessed when sufficient data have accumulated. In the absence of these data, the presence of characteristic patterns of brain activity in mammals during NREM and REM sleep, alongside the cognitive 'black-out' that is experienced while sleeping, imply that it does perform some important function for the brain. The nature of this function remains hotly debated [1]

An important implication of our findings is that ecological factors that impact sleep could indirectly affect immune defences. By sleeping regularly in 'safe' sites such as burrows or dens, for example, individuals may be better protected from predators, and thus able to sleep for longer durations [2-4,45]. Conversely, herbivorous species with large foraging requirements could have less time available for sleep than species living on an energy-rich carnivorous diet [2,4,5]. Trade-offs between time invested in sleep and alternative activities may occur at key life history stages, such as during periods of reproductive competition [6] or parental care [46]. Current evidence links these activities to reduced immunocompetence [47], which could be due to reductions in the time available for sleep. However, field studies should also ascertain the relationship between sleeping behaviour and an animal's exposure to parasites, which could reveal important ecological relationships between sleep and parasitic infection that have the potential to influence the analyses we report here [48].

## Conclusion

Our results suggest mammalian species that spend more time asleep are able to increase investment in their immune systems, and thus are better protected from parasitic infection. These comparative findings are broadly consistent with experimental evidence showing close physiological links between sleep and the immune system, and point towards a major role for disease resistance in the evolution of mammalian sleep. Given the declines in human sleep durations that have occurred in recent decades [49], there is a clear need for studies that further clarify the immunological significance of sleep. In particular, studies using artificial selection regimes would allow the evolutionary relationship between immunocompetence and sleep to be quantified under controlled experimental conditions, and without potential confounds that are associated with sleep-deprivation studies [50] and the correlational analyses we present here. Similarly, further studies assessing the selective advantages of modulating sleep durations and its sub-phases during infection are clearly warranted. There is also a need to further uncover the physiological mechanisms underpinning the influence of sleep on the immune system, to examine how ecological factors might constrain sleeping durations, and to investigate whether sleep deficits increase susceptibility to disease at key stages of an animal's life history.

## Methods

### Data collection

Species specific sleep times were compiled from an exhaustive search of the published literature [see Additional File 1 for details]. Data were screened for quality and included in analyses if the study animals were sexually mature (according to reference [51]), and the study was designed to capture the entire sleeping period. This generally entailed observation periods of at least 24 hours, though overnight observations on monophasic diurnal primate species (lasting 12 to 14 hours) were included in five cases. Where multiple estimates of sleep durations were available for a given species, we calculated an average value for use in analyses.

For marine mammals, estimates were for animals sleeping on land and, following others (e.g. [10]), unihemispheric sleep times for the left and right sides of the brain were combined by averaging them. As the energetic cost of maintaining half of the brain in a waking state is unclear, we also tested alternative methods for the calculation of unihemispheric sleeping times (combining or excluding time spent in unihemispheric sleep), but this did not produce qualitative differences in our results (unpublished analyses).

We use behavioural studies of sleep in our analyses of total sleep durations (n = 7), but restrict our analyses of NREM and REM sleep durations with haematological parameters to studies that utilized electroencephalographic (EEG) recordings of sleep [see Additional File 2 for an analysis of parasite counts and sleep that has been similarly restricted to studies that used EEG measurement of sleep durations]. The duck billed platypus (*Ornithorhynchus anatinus*) was excluded from analyses of NREM and REM sleep, as the validity of REM sleep times for this species has been questioned [52].

The across-study repeatability of sleeping time estimates using these criteria was high (0.82, F_15,24 _= 12.67, [53]; data are from a larger sleep dataset [see Additional File 1]), indicating that studies using our data selection criteria recorded very similar sleep durations for a given species. Thus, further restriction of the dataset (e.g. to EEG only data [3,4]) would appear unnecessary for comparative analyses, as any remaining measurement error is small relative to the wide variation in sleep durations between different species. Overall, this dataset compares favourably with studies that have utilized all available sleep data (r = 0.42), and a previous analysis of ours that had used alternate restrictions to control data quality (r = 0.71 [4]).

There remains a possibility that data on captive animals represent overestimates of sleeping times in the wild, as in a more natural setting animals would seem likely to experience ecological constraints on the time available for sleep (e.g. predator avoidance [3]). While behavioural studies on shrews (*Sorex araneus*) that compared sleep times in the wild and in the laboratory recorded similar sleep durations [54], a more recent EEG based study on the sloth (*Bradypus tridactylus*) suggested that this species may sleep less in the wild than had been recorded in the laboratory [55,56]. However, this comparison included laboratory estimates from juvenile animals that are known to sleep for longer durations, and is therefore questionable [55-57]. More generally, the occurrence of 'sleep rebounds' in a laboratory setting, in which animals sleep more following a period of sleep deprivation, suggest that the sleeping times that have been recorded are homeostatically regulated and therefore representative of an animal's daily sleep requirement [58].

Haematological data were extracted from the physiological reference values reported by ISIS [24], and are from zoo animals that are judged to be in normal health. These reference values are designed to be used in a diagnostic capacity by veterinary professionals, and are screened for anomalous data. On average, these data have arisen from blood samples derived from 20 different zoological institutions (range: 1 to 52), with 76 animals contributing to the reference value (range: 4 to 289). Insufficient data were available in some species to separate cell counts by age or sex; however, repeatability between sex and age groups were high (for the white blood cell counts of species present in analyses: 0.88, F_15,16 _= 16.51, and 0.7, F_15,29 _= 7.76, respectively [53]). Thus, we included averaged values in analyses from animals of mixed sex and age class.

Data on parasitism were extracted from the *Global Mammal Parasite Database *[28]. This database summarizes studies that describe patterns of parasite occurrence in wild populations and includes both macro- and microparasites. The data were restricted to studies that provided measures of prevalence. Relative infection status was calculated as the number of parasite species found in species multiplied by the (mean) percentage of animals infected. By taking account of the prevalence of parasite species, this measure avoids giving undue weight to parasites that rarely infect a particular host species successfully.

### Data analysis

Phylogenetically independent contrasts were calculated using the CRUNCH algorithm in the CAIC computer program [59], with branch lengths set to be equal. The assumptions of independent contrasts were best met with a combination of natural logarithm, square root, and reciprocal square root transformations [see Additional File 1 for specific details [60]]. Analyses were performed using multiple or univariate regression, with the regression line forced through the origin [59,60]. Bootstrap estimates are presented when there was evidence of variance heterogeneity or outlying data-points [61]. Regression analyses were implemented in Genstat 8^th ^edition and controlled for an influence of body mass, activity period, and sampling effort when appropriate (see next section). Significance of variables is judged from the estimate of the regression coefficient in association with its standard error; the P values presented are calculated from the t statistic that is derived from these estimates and the sample size of each analysis. For predictions and plots, activity period was set to be diurnal, while body mass and sampling effort were held at average levels.

### Statistical control for body mass

Previous comparative studies have identified allometric scaling relationships in haematological parameters [62,63]. We found a similar influence of body mass in our analyses of white blood cell counts using the raw data (coefficient = 0.0257, s.e. = 0.00564, t_22 _= 4.55, *P *< 0.001), white blood cell counts using phylogenetically independent contrasts (coefficient = 0.0177, s.e. = 0.005, t_23 _= 3.55, *P *= 0.002), and the relative abundance of white blood cells (coefficient = 0.228, s.e. = 0.053, t_20 _= 4.29, *P *< 0.001). We also found a negative relationship between body mass and red blood cell counts (contrast analysis, coefficient = -1.793, s.e. = 0.566, t_23 _= -3.17, *P *= 0.004). For white blood cell counts, these relationships appeared to be driven by the allometric scaling of neutrophils and monocytes (bootstrapped estimates for: neutrophils, coefficient = 0.1227, s.e. = 0.0503, *P *= 0.002; monocytes, coefficient = 0.1099, s.e. = 0.0557, *P *< 0.05; for all other cell types *P *> 0.7). In each of these cases, the results we present are therefore derived from multiple regressions in which body mass was included as an explanatory variable.

### Statistical control for activity period

White blood cell counts exhibit cyclical diurnal variation [64,65] that is intimately linked to the sleep-wake cycle [64]. We therefore examined whether the different activity period of nocturnal species was reflected in their white blood cell counts. We found that nocturnal species had significantly reduced white blood cell counts (nocturnality: effect = -0.0300, s.e. = 0.0120, t_22 _= -2.50, *P *= 0.02), which appeared to be driven solely by changes in neutrophil numbers (for log transformed neutrophil counts: effect = -0.544, s.e. = 0.159, t_22 _= -3.42, *P *= 0.002; in analyses of all other cell types activity period was not significant, *P *> 0.33). This effect of nocturnality on neutrophil numbers and total white blood cell count was statistically corrected prior to the generation of independent contrasts; effect sizes were obtained from the parameter estimates in the corresponding multiple regression and are listed above. Analyses of white blood cells that did not account for differences in activity periods produced qualitatively similar results [see Additional File 2].

### Statistical control for sampling effort

Estimates of the number of micro- and macroparasites that infect host species are dependent upon the degree to which the host species has been studied [29-31]. Thus, we statistically corrected our estimates of infection status prior to analysis by controlling for the highly significant influence of sampling effort (estimated by the number of studies that described the parasites of each host; raw species data: coefficient = 1.43, s.e. = 0.284, t_10 _= 4.03, P = 0.002; contrasts: coefficient = 1.275, s.e. = 0.286, t_10 _= 4.45, P = 0.001). Citation number explained 58% and 64% of the variance in species and contrast data on parasitism, respectively.

## Supplementary Material

Additional file 1 **Data on sleep, immunity and infection.** Species specific values for the time spent in sleep and its different states, the number of white blood cells in peripheral blood, and the degree of parasitism. Click here for file

Additional file 2 **Statistical analyses with alternate data restrictions.** Alternate analyses in which the relationship between sleep and parasitism is restricted to EEG studies, and in which the relationship between sleep and immune defence does not control for activity period. Click here for file
