# Supplementary material for: Parasite resistance and the adaptive significance of sleep
Source: BMC Evol Biol. 2009 Jan 9;9:7. doi: 10.1186/1471-2148-9-7 (PMC2631508; doi:10.1186/1471-2148-9-7)
Supplement: Additional file 1 — Data on sleep, immunity and infection. Species specific values for the time spent in sleep and its different states, the number of white blood cells in peripheral blood, and the degree of parasitism. [file 1471-2148-9-7-S1.pdf]

Table 1. Sleeping times, white blood cell counts, and associated variables in 26 mammalian species.

Species	Total Sleep†	NREM Sleep†	REM Sleep†	Total white cell count‡	Basophils‡	Eosinophils‡	Lymphocytes‡	Monocytes‡	Neutrophils‡	Red blood cells‡	Platelets‡	Body Mass¶	Nocturnal [71, 72]
Aotus trivirgatus	16.97 [73]	15.15 [73]	1.82 [73]	10.580	0.206	1.768	4.829	0.292	4.134	5.66	0.255	-0.256 [74]	Y
Bos Taurus	3.94 [75]	3.19 [75]	0.74 [75]	9.471	0.056	0.371	4.432	0.462	4.406	8.20	*	6.383 [76, 77]	N
Canis familiaris	9.23 [78-80]	6.42 [78-80]	2.81 [78-80]	10.360	0.222	0.610	2.627	0.448	6.695	6.60	0.287	3.039 [76, 77, 81-84]	N
Cavia porcellus	6.82 [85]	5.88 [85]	0.94 [85]	8.741	0.081	0.555	4.981	0.455	3.026	5.07	*	-0.608 [77, 86]	N
Cercopithecus aethiops	9.77 [87]	9.04 [87]	0.73 [87]	7.431	0.063	0.343	2.652	0.769	3.916	5.52	0.287	1.444 [74]	N
Chinchilla lanigera	12.53 [88]	10.98 [88]	1.55 [88]	6.528	0.120	0.310	3.926	0.224	2.149	7.16	0.357	-0.801 [89-91]	Y
Choloepus hoffmanni	16.50 [92]	*	*	13.280	0.416	0.495	7.737	0.700	4.25	3.11	0.277	1.573 [76]	Y
Elephas maximus	3.51 [93]	*	*	14.44	0.180	0.604	5.296	3.665	4.737	3.07	0.432	8.011 [89, 94]	N
Equus caballus	2.88 [75]	2.09 [75]	0.79 [75]	7.694	0.087	0.220	3.110	0.299	4.092	7.44	0.218	5.927 [76, 77, 89, 95]	N
Erinaceus europaeus	17.64 [96]	14.03 [96]	3.62 [96]	15.100	0.360	1.142	5.287	0.409	7.979	6.80	0.196	-0.088 [77, 89, 97]	Y
Felis catus	12.64 [98, 99]	9.04 [98, 99]	3.60 [98, 99]	10.480	0.128	0.676	3.810	0.325	5.728	8.59	0.25	1.144 [76, 77, 89]	N
Macaca mulatta	10.23 [100, 101]	8.19 [100, 101]	2.05 [100, 101]	9.969	0.119	0.467	4.872	0.292	4.772	5.42	0.468	2.088 [74]	N
Octodon degus	6.85 [102, 103]	8.21 [103]	0.82 [103]	5.065	0.117	0.309	2.852	0.103	2.013	7.48	0.3	-1.575 [89, 90, 104, 105]	N
Ornithorhynchus anatinus	14.20 [106]	8.20 [106]	5.40 [106]	30.930	0.333	0.763	18.620	2.459	9.354	11.30	*	0.601 [89, 107]	N
Oryctolagus cuniculus	8.45 [108]	7.35 [108]	1.09 [108]	6.152	0.179	0.252	3.145	0.307	2.438	5.69	0.355	0.479 [77, 89]	Y
Ovis aries	3.84 [75]	3.26 [75]	0.58 [75]	6.622	0.073	0.301	3.273	0.239	2.921	10.08	0.443	3.760 [5]	N

Pan troglodytes	9.67 [109]	8.22 [109]	1.45 [109]	10.800	0.082	0.240	3.173	0.379	7.039	5.49	0.248	3.806 [74]	N
Panthera onca	10.40 [110]	*	*	11.770	0.074	0.316	2.096	0.345	8.896	7.26	0.281	4.340 [89, 111]	Y
Papio hamadryas	9.83 [112]	8.57 [112]	1.267 [112]	11.550	0.051	0.158	2.143	0.333	8.998	5.27	0.406	2.694 [74]	N
Petterus macaco	9.64 [113]	8.81 [113]	0.84 [113]	9.128	0.114	0.476	3.872	0.296	4.638	8.46	0.205	0.599 [74]	N
Petterus mongoz	11.88 [113]	11.16 [113]	0.72 [113]	7.204	0.093	0.412	3.494	0.253	3.045	9.70	0.653	0.396 [74]	N
Rattus norvegicus	13.13 [85, 88, 114, 115]	10.89 [85, 88, 114, 115]	2.05 [85, 88, 114, 115]	10.390	0.063	0.224	7.749	0.781	1.560	6.97	0.336	-1.255 [76, 77, 89]	Y
Saguinus oedipus	13.18 [116]	*	*	10.960	0.117	0.214	3.389	0.627	6.783	6.26	0.325	-0.889 [74]	N
Saimiri sciureus	8.72 [116, 117]	8.2 [117]	1.4 [117]	8.623	0.097	0.272	3.349	0.358	4.449	6.98	0.326	-0.235 [74]	N
Theropithecus gelada	10.92 [118]	*	*	9.173	0.093	0.244	3.665	0.344	5.130	4.50	0.376	2.731 [74]	N
Vulpes vulpes	9.72 [119]	7.39 [119]	2.40 [119]	5.782	0.109	0.613	1.771	0.214	3.167	12.14	0.392	1.474 [89]	Y

The data used to assess the relationship between sleep and immune investment while controlling for differences in body size and activity period. Numbers in brackets denote the data source(s), see the supporting references; † Hours per day; ‡ ISIS physiological references values (white blood cell counts x10^9/L, red blood cell counts x10^{12}/L, platelets x10^{12}/L); ¶ log transformed. For contrast analyses: Total white blood cell count, lymphocyte count and monocyte counts were reciprocal square root transformed; neutrophils, basophils, NREM and REM sleep were log transformed; eosinophils were square root transformed. The full Phylogeny of Sleep Database can be found at: http://www.bu.edu/phylogeny/index.html.

Table 2. Sleeping times and parasite susceptibility in 12 mammalian species.

Species	Total sleep[†]	Number of parasites[‡]	Prevalence[‡] (%)	Citation count[‡]
Aotus trivirgatus	16.97 [73]	4	2.87	4
Arctocephalus pusillus	6.37 [120]	5	32.20	1
Callorhinus ursinus	7.30 [121]	2	31.90	2
Cercopithecus aethiops	9.77 [87]	8	30.28	9
Macaca radiata	9.12 [122, 123]	1	69.00	1
Pan troglodytes	9.67 [109]	15	24.69	12
Papio anubis	9.20 [124]	33	23.60	19
Papio papio	10.19 [112, 123, 125]	2	11.00	2
Saguinus oedipus	13.18 [116]	1	20.00	1
Saimiri sciureus	8.72 [116, 117]	7	15.47	10
Theropithecus gelada	10.92 [118]	1	6.00	1
Vulpes vulpes	9.72 [119]	74	17.30	72

The data used to assess the relationship between sleep and parasitic infection while controlling for differences in sampling effort. Numbers in brackets denote the data source(s), see the supporting references; [†] Hours per day, [‡]As reported by the *Global Mammal Parasite Database*. Full details of the database can be found at: http://www.mammalparasites.org/. Variables were log-transformed for analyses.

References

71. Footit TL: **Mammalian activity timing. A preliminary comparative study**. Durham, UK: Durham University; 1999.
72. Nowak RM: **Walker's mammals of the world**. London: John Hopkins University Press; 1999.
73. Perachio AA: **Sleep in the nocturnal primate, *Aotus trivirgatus*.** *Proc 3rd Int Cong Primatol* 1970, **2**:54-60.
74. Smith RJ, Jungers WL: **Body mass in comparative primatology**. *Journal of Human Evolution* 1997, **32**(6):523-559.
75. Ruckebusch Y: **The relevance of drowsiness in the circadian cycle of farm animals.** . *Anim Behav* 1972, **20**(4):637-643.
76. Crile G, P. QD: **A record of the body weight and certain organ and gland weights of 3690 animals.** *Ohio J Sci* 1940, **40**:219-259.
77. Leitch I, Hytten FE, Billewicz WZ: **The maternal and neonatal weights of some mammalia.** *Proc Zool Soc Lond* 1959, **133**:11-29.
78. Copley M, Jennings D, Mitler M: **A study of continuous forty-eight hour sleep-waking recordings in five dogs.** *Sleep Res* 1976, **5**:94.
79. Lucas EA, Powell EW, Murphree OD: **Baseline sleep-wake patterns in the pointer dog.** *Physiol Behav* 1977, **19**:285-291.
80. Takahashi Y, Hoinhara S, Nakamura Y, Takahashi K: **A model of human sleep-related growth hormone secretion in dogs: Effects of 3, 6, and 12 hours of wakefulness on plasma growth hormone, cortisol, and sleep stages.** *Endocrinology* 1981, **109**(1):262-272.
81. Dore MAP: **Structural aspects of luteal function and regression in the ovary of the domestic dog.** *J Reprod Fert Suppl* 1989, **39**:41-53.
82. Shille VM, Thatcher MJ, Lloyd ML, Miller DD, Seyfert DF, Sherrod JD: **Gonadotrophic control of follicular development and the use of exogenous gonadotrophins for induction of oestrus and ovulation in the bitch.** *J Reprod Fertil Suppl* 1989, **39**:103-113.
83. Concannon PW: **Induction of fertile oestrus in anoestrous dogs by constant infusion of GnRH agonist.** *J Reprod Fert Suppl* 1989, **39**:149-160.
84. Gilbert RO, Nothling JO, Oettle EE: **A retrospective study of 40 cases of canine pyometra-metritis treated with prostaglandin F-2 alpha and broad-spectrum antibacterial drugs.** *J Reprod Fertil Suppl* 1989, **39**:225-229.
85. Pellet J, Beraud C: **The circadian sleep-wakefulness organization of the guinea pig (*Cavia porcellus*).** *Physiol Behav* 1967, **2**:131-137.
86. von Bonin G: **Brain weight and body weight in mammals.** *J Gen Psychol* 1937, **16**:379-389.
87. Balzamo E, Vuillon-Cacciuttolo G, Bert J: ***Cercopithecus aethiops*: EEG et organization des etats de vigiliance.** *Wake Sleep* 1978, **2**:223-230.
88. Van Twyver H: **Sleep patterns in five rodent species.** *Physiol Behav* 1969, **4**:901-905.
89. Silva M, Downing JA: **CRC handbook of mammalian body masses.** Boca Raton, Florida: CRC Press Inc.; 1995.
90. Rezende EL, Bozinovic F, Garland T: **Climatic adaptation and the evolution of basal and maximum rates of metabolism in rodents.** *Evolution* 2004, **58**(6):1361-1374.
91. Spotorno AE, Zuleta CA, Valladares JP, Deane AL, Jiménez JE: ***Chinchilla laniger*.** *Mammal Spec* 2004, **758**:1-9.
92. Sunquist M, Montgomery G: **Activity patterns and rates of movement of two-toed and three-toed sloths, *Choloepus hoffmani* and *Bradypus infuscatus*.** *J Mammal* 1973, **54**:946-954.
93. Tobler I: **Behavioral sleep in the Asian elephant in captivity.** *Sleep* 1992, **15**(1):1-12.
94. Shoshani J, Eisenberg JF: ***Elephas maximus*.** *Mammal Spec* 1982, **182**:1-8.
95. Tischner M: **Development of Polish pony foals born after embryo transfer to large mares.** *J Reprod Fert Suppl* 1987, **35**:705-709.
96. Fourre AM, Rodriquez FL, Vincent JD: **Etude polygraphique de la veille et du sommeil chez le herisson (*Erinaceus europaeus* L.).** *C R Soc Biol Fil (Paris)* 1974, **168**:959-964.
97. Bauchot R, Stephan H: **Donnees nouvelles sur l'encephalisation des insectivores et des prosimiens.** *Mammalia* 1966, **30**:160-196.
98. Lucas EA: **Effects of a short light-dark cycle on the sleep-wake patterns of the cat.** *Sleep* 1979, **1**(3):299-317.
99. Lucas EA, Sterman MB: **The polycyclic sleep-wake cycle in the cat: Effects produced by sensorimotor rhythm conditioning.** *Exp Neurol* 1974, **42**:347-368.
100. Balzamo E, Van Beers P, Lagarde D: **Scoring of sleep and wakefulness by behavioral analysis from video recordings in rhesus monkeys: Comparison with conventional EEG analysis.** . *Electroencephalogr & Clin Neurophysiol* 1998, **106**:206-212.
101. Robinson EL, Hsieh JK, Fuller CA: **A primate model of sleep regulation.** *Sleep Suppl* 2003, **26**:A391.

102. Fischer RB, Meunier GF: **Effects of enclosure size on activity and sleep of a hystricomorph rodent (*Octodon degus*).** *Bull Psychon Soc* 1980, **16**(4):273-275.
103. Kas MJ, Edgar DM: **Crepuscular rhythms of EEG sleep-wake in a hystricomorph rodent, *Octodon degus*.** *J Biol Rhythms* 1998, **13**(1):9-17.
104. Woods CA, Boraker DK: ***Octodon degus*.** *Mammal Spec* 1975, **67**:1-5.
105. Bozinovic F, Bacigalupe LD, Vasquez RA, Visser GH, Veloso C, Kenagy GJ: **Cost of living in free-ranging degus (*Octodon degus*): seasonal dynamics of energy expenditure.** *Comp Biochem Physiol A Mol Integr Physiol* 2004, **137**(3):597-604.
106. Siegel JM, Manger PR, Nienhuis R, Fahringer HM, Shalita T, Pettigrew JD: **Sleep in the platypus.** *Neuroscience* 1999, **91**(1):391- 400.
107. Rose RW, Nevison CM, Dixson AF: **Testes weight, body weight and mating systems in marsupials and monotremes.** *J Zool* 1997, **243**:523-531.
108. Pivik RT, Bylsma FW, Cooper P: **Sleep-wakefulness rhythms in the rabbit.** *Behav Neural Biol* 1986, **45**(3):275-286.
109. Bert J, Kripke DF, Rhodes J: **Electroencephalogram of the mature chimpanzee: Twenty-four hour recordings.** *Electroencephalogr & Clin Neurophysiol* 1970, **28**(32-40).
110. Zepelin H: **Sleep of the jaguar and the tapir: A prey-predator contrast.** *Psychophysiology* 1970, **7**(305-306).
111. Seymour KL: ***Panthera onca*.** *Mammal Spec* 1989, **340**:1-9.
112. Bert J: **Similarities and differences in the sleep of two baboons, *Papio hamadryas* and *Papio papio*.** *Electroencephalogr & Clin Neurophysiol* 1973, **35**:209-212.
113. Balzamo E, Vuillon-Cacciuttolo G, Petter J, Bert J: **Etats de vigiliance chez deux Lemuridae: Rhythmes EEG et organization obtenus par telemesure.** *Wake Sleep* 1978, **2**:237-245.
114. Kiyono S: **Sleep studies in gunn rats.** *Sleep Res* 1975, **4**:227.
115. Mistlberger RE, Bergmann BM, Waldenar W, Rechtschaffen A: **Recovery sleep following sleep deprivation in intact and suprachiasmatic nuclei-lesioned rats.** . *Sleep* 1983, **6**:217-233.
116. Kantha SS, Suzuki J: **Sleep quantitation in common marmoset, cotton top tamarin and squirrel monkey by non-invasive actigraphy.** *Comp Biochem Physiol A* 2006, **144**:203-210.
117. Wexler DB, Moore-Ede MC: **Circadian sleep-wake cycle organization in squirrel monkeys.** *Am J Physiol Regul Integr & Comp Physiol* 1985, **248**:R353-R362.
118. Noser R, Gygax L, Tobler I: **Sleep and social status in captive gelada baboons (*Theropithecus gelada*).** . *Behav Brain Res* 2003, **147**(1-2):9-15.
119. Dallaire A, Ruckebusch Y: **Rest-activity cycle and sleep patterns in captive foxes (*Vulpes Vulpes*).** *Experientia* 1974, **30**:59-60.
120. Lyamin OI, Chetyrbok IS: **Unilateral EEG activation during sleep in the Cape fur seal, Arctocephalus pusillus.** *Neurosci Lett* 1992, **143**((1-2)):263-266.
121. Lyamin OI, Mukhametov LM, Polyakova IG: **Peculiarities of sleep in water in northern fur seals**. *Zh Vyssh Nerv Deiat Im I P Pavlova* 1986, **36**((6)):1039-1044.
122. Bert J, Pegram V, Balzamo E: **Comparison du sommeil de deux macaques (Macaca radiata et Macaca mulatta)**. *Folia Primatol* 1972, **17**:202-208.
123. Bert J: **Adaptation du sommeil aux conditions experimentales d'enregistrement chez deux Cercopithecinae (Papio papio et Macaca radiata)**. *Proc 3rd Int Cong Primatol* 1970, **2**:49-53.
124. Balzamo E, Bert J: **Sleep in Papio anubis: Its organization and lateral geniculate spikes.** *Sleep Res* 1975, **4**:138.
125. Bert J, Balzamo E, Chase M, Pegram V: **The sleep of the baboon, Papio papio, under natural conditions and in the laboratory.** *Electroencephalogr & Clin Neurophysiol* 1975, **39**:657-662.
