# Supplementary material for: Parasite resistance and the adaptive significance of sleep
Source: BMC Evol Biol. 2009 Jan 9;9:7. doi: 10.1186/1471-2148-9-7 (PMC2631508; doi:10.1186/1471-2148-9-7)
Supplement: Additional file 2 — Statistical analyses with alternate data restrictions. Alternate analyses in which the relationship between sleep and parasitism is restricted to EEG studies, and in which the relationship between sleep and immune defence does not control for activity period. [file 1471-2148-9-7-S2.pdf]

Additional File 2

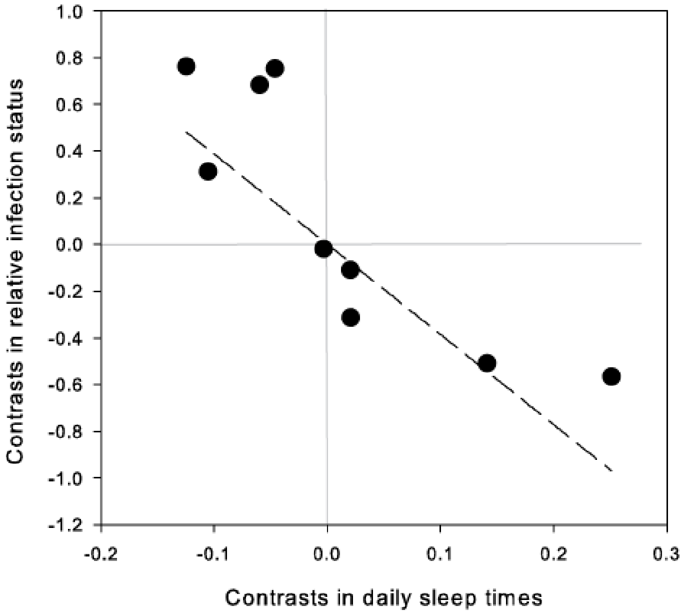

Figure 3. Sleep and parasitism restricted to EEG studies of sleep.

A plot of independent contrasts showing relative infection status as a function of daily sleep times. Restricting our analyses to include only EEG studies of sleep does not qualitatively affect our results; regression coefficient = -3.670, s.e. = 0.950, t_8 = 3.86, P = 0.005.

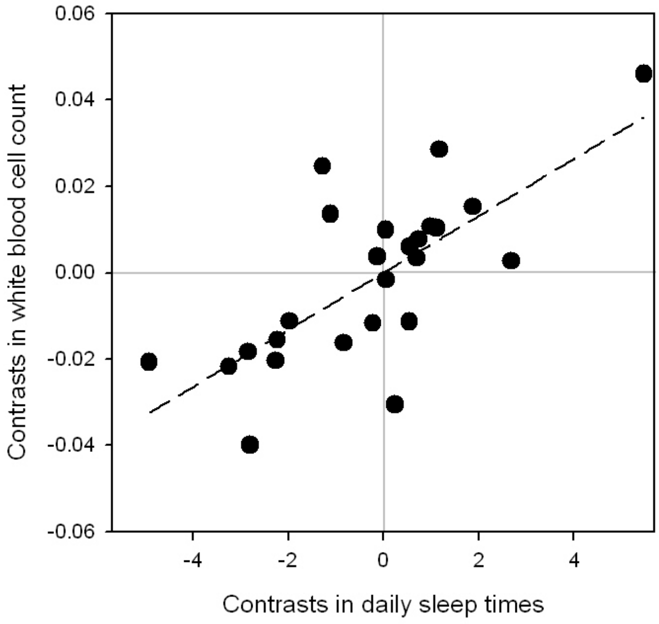

Figure 4. Sleep and immune defence without control for activity period. A plot of independent contrasts showing white blood cell counts as a function of daily sleep times; regression coefficient = 0.00655, s.e. = 0.00186, t_{23} = 3.52, P = 0.002. The plot controls for a significant association between white blood cell counts and body size; regression coefficient = 0.00979, s.e. = 0.00372, t_{23} = 2.63, P = 0.015.
